# Superficial esophageal squamous cell carcinoma with melanocytosis that was endoscopically difficult to differentiate from malignant melanoma

**DOI:** 10.1002/deo2.353

**Published:** 2024-03-25

**Authors:** Yoshinori Horikawa, Kenichi Goda, Ryota Koyanagi, Masayuki Kondo, Keiichiro Abe, Akira Kanamori, Koichi Hamada, Kazuyuki Ishida, Atsushi Irisawa

**Affiliations:** ^1^ Department of Gastroenterology Dokkyo Medical University Tochigi Japan; ^2^ Department of Gastroenterology Southern‐Tohoku General Hospital Fukushima Japan; ^3^ Department of Gastroenterology Utsunomiya Memorial Hospital Tochigi Japan; ^4^ Department of Minimally Invasive Surgical and Medical Oncology Fukushima Medical University Fukushima Japan; ^5^ Department of Diagnostic Pathology Dokkyo Medical University Tochigi Japan

**Keywords:** biopsy, endoscopic submucosal dissection, esophageal squamous cell carcinoma, melanocytosis, malignant melanoma

## Abstract

Esophageal squamous cell carcinoma (SCC) with dark spots caused by melanocytosis is very rare. A reddish and flat lesion, 4 cm in length and covering over two‐thirds of the circumference, was found in the midthoracic esophagus of a 66‐year‐old male. Multiple brown and black spots are observed in the lesion. Superficial SCC with melanocytosis or malignant melanoma was also suspected. Endoscopic submucosal dissection was performed without biopsies of the spots. Histologically, a few melanocytes were observed in the black spots, and the lesion was diagnosed as SCC (T1a‐lamina propria mucosae) with melanocytosis. We report a case of esophageal SCC with dark black spots that were difficult to differentiate endoscopically from malignant melanoma.

## INTRODUCTION

Dark‐colored esophageal lesions have been suggested to be caused by melanocytosis and malignant melanoma. Esophageal squamous cell carcinoma (SCC) accompanied by dark spots caused by melanocytosis is extremely rare.^1^ Our search uncovered only one report of three lesions (one case each of intraepithelial neoplasia, atypical squamous cell proliferation, and SCC in situ with melanocytosis).^1^ We report a case of esophageal SCC with dark black spots that were difficult to differentiate endoscopically from malignant melanoma.

## CASE REPORT

A 66‐year‐old male underwent esophagogastroduodenoscopy (as part of a routine health examination. His previous physician detected a flat reddish lesion with dark brown and black spots in the mid‐thoracic esophagus. Biopsy of the lesion away from the dark spots revealed SCC.

The possibility of SCC being complicated by malignant melanoma rather than melanocytosis was considered by a previous doctor due to the presence of dark black spots. The patient was then referred to our hospital for further examination and treatment. The patient had no history of smoking, but he had a history of alcoholic beverage consumption of 500 mL of beer per day, 6 days per week, for 46 years and responded positively to the alcohol flush reaction. Blood count and biochemistry tests, including those for tumor markers, showed no abnormal findings. Contrast‐enhanced computed tomography of the chest and abdomen did not confirm a primary tumor in the mid‐thoracic esophagus, and there were no findings suggestive of lymph node or distal metastasis. No pigmentation was observed on the eyeballs, anus, rectal mucosa, or anywhere on the skin.

**FIGURE 1:**
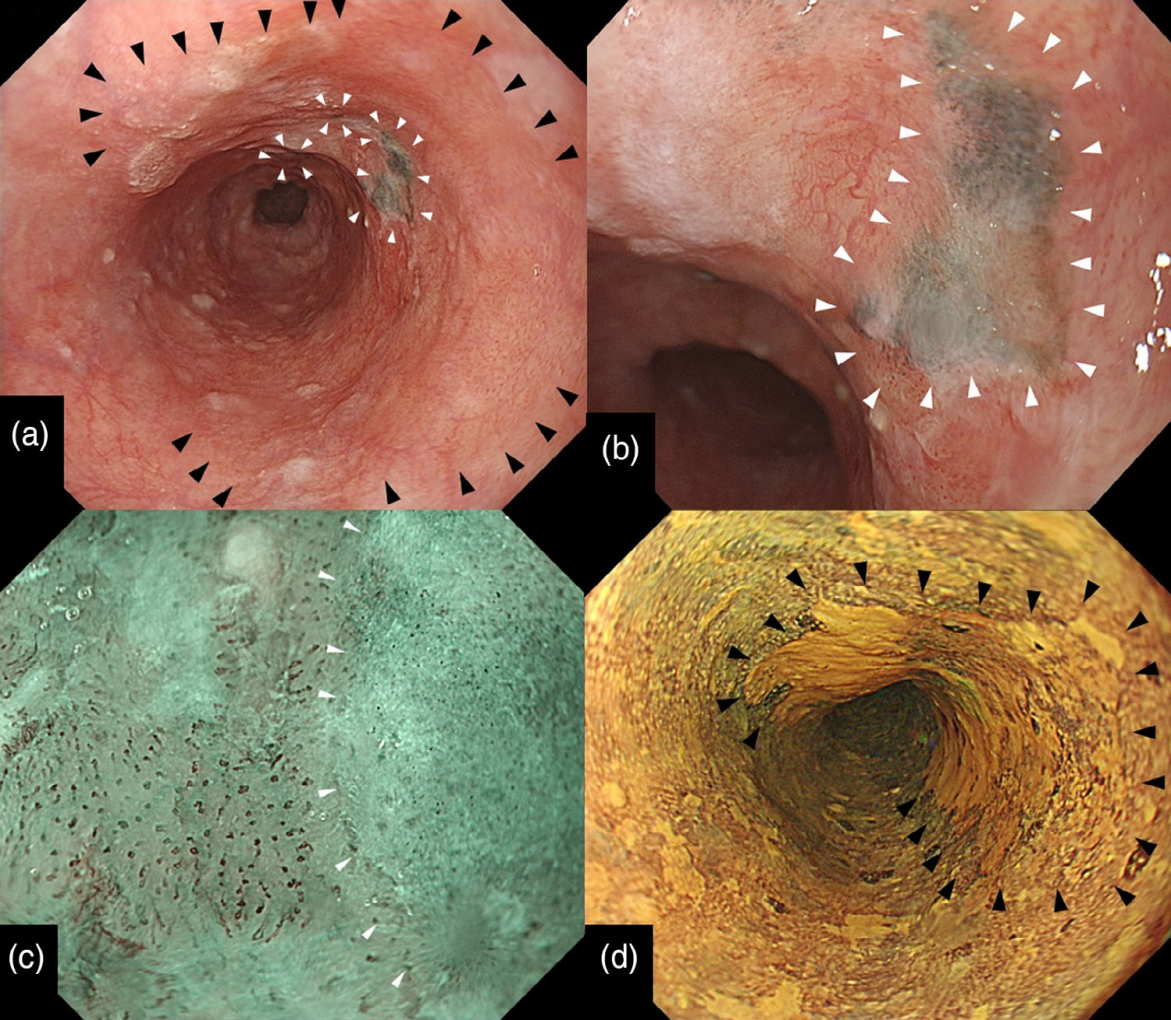
Endoscopic images of the esophagus. (a) A white light image showed a flat lesion (0–IIb) presenting with redness over two‐thirds of its circumference (black arrowheads). Three spots were dark brown to black with irregular and well‐defined borders within the lesion (white arrowheads). (b) The black spot showed slight thickening and well‐defined borders (white arrowheads). (c) Narrow band imaging magnifying endoscopy indicated that the reddish lesion had type B1 vessels according to the Japanese Esophageal Society Magnifying Endoscopy Classification. However, vessels were not visible in the dark brown to black spots (white arrowheads). Vessels were not visible in the dark brown to black spots. (d) Lugol chromoendoscopy showed the entire lesion was 4 cm long and was a region covering two‐thirds of its circumference (arrowheads).

**FIGURE 2:**
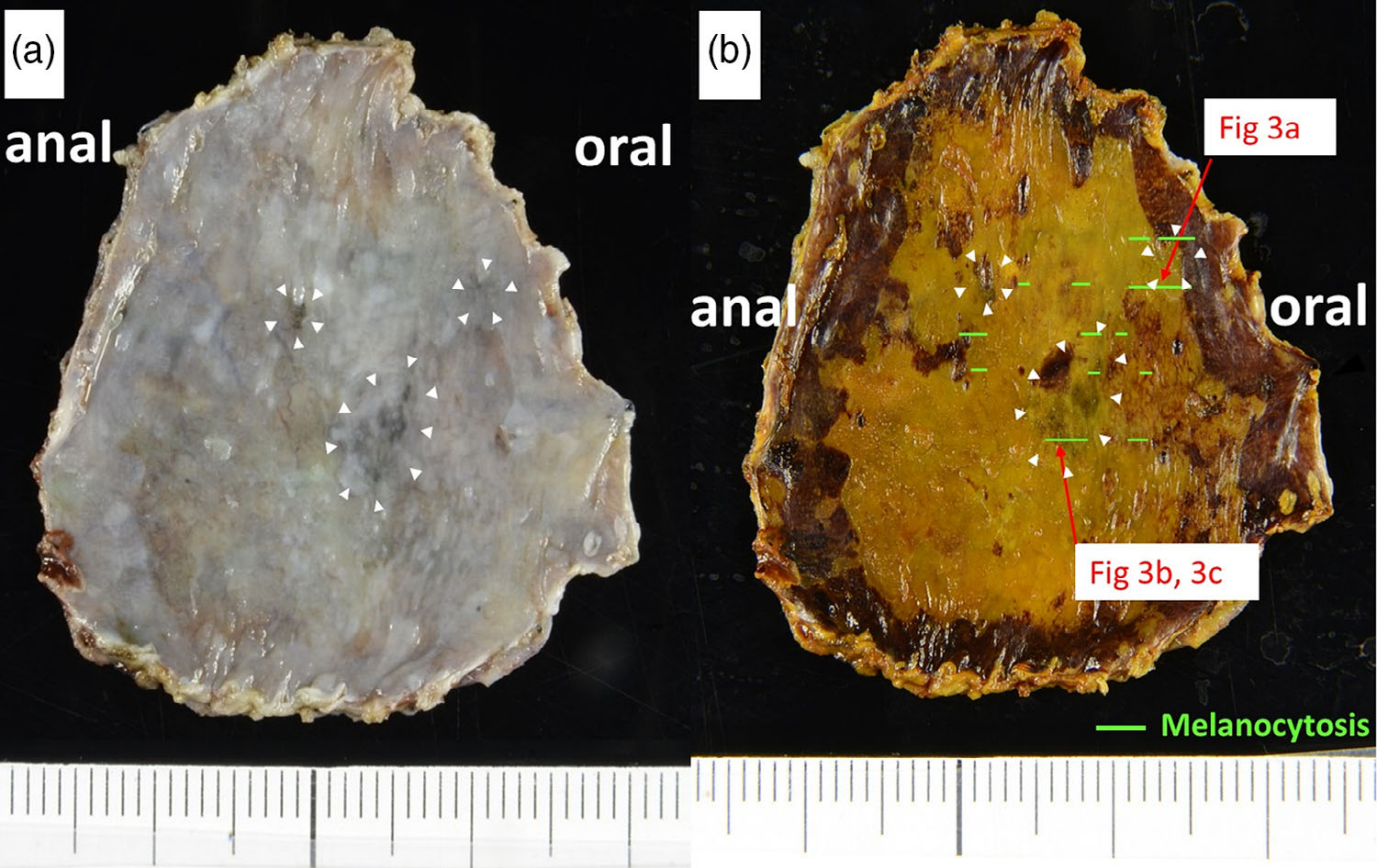
Endoscopic submucosal dissection specimen. (a) Macroscopic finding. There were black spots with well‐defined borders in the lesion (white arrowheads). (b) The endoscopic submucosal dissection specimen measured 42 × 30 mm^2^, with a flat Lugol voiding lesion measuring 32 × 30 mm^2^. Arrows within Figure 2 indicated a mapping of the histological images corresponding to Figure 3a–c. Areas of melanocytosis are indicated by green lines.

**FIGURE 3:**
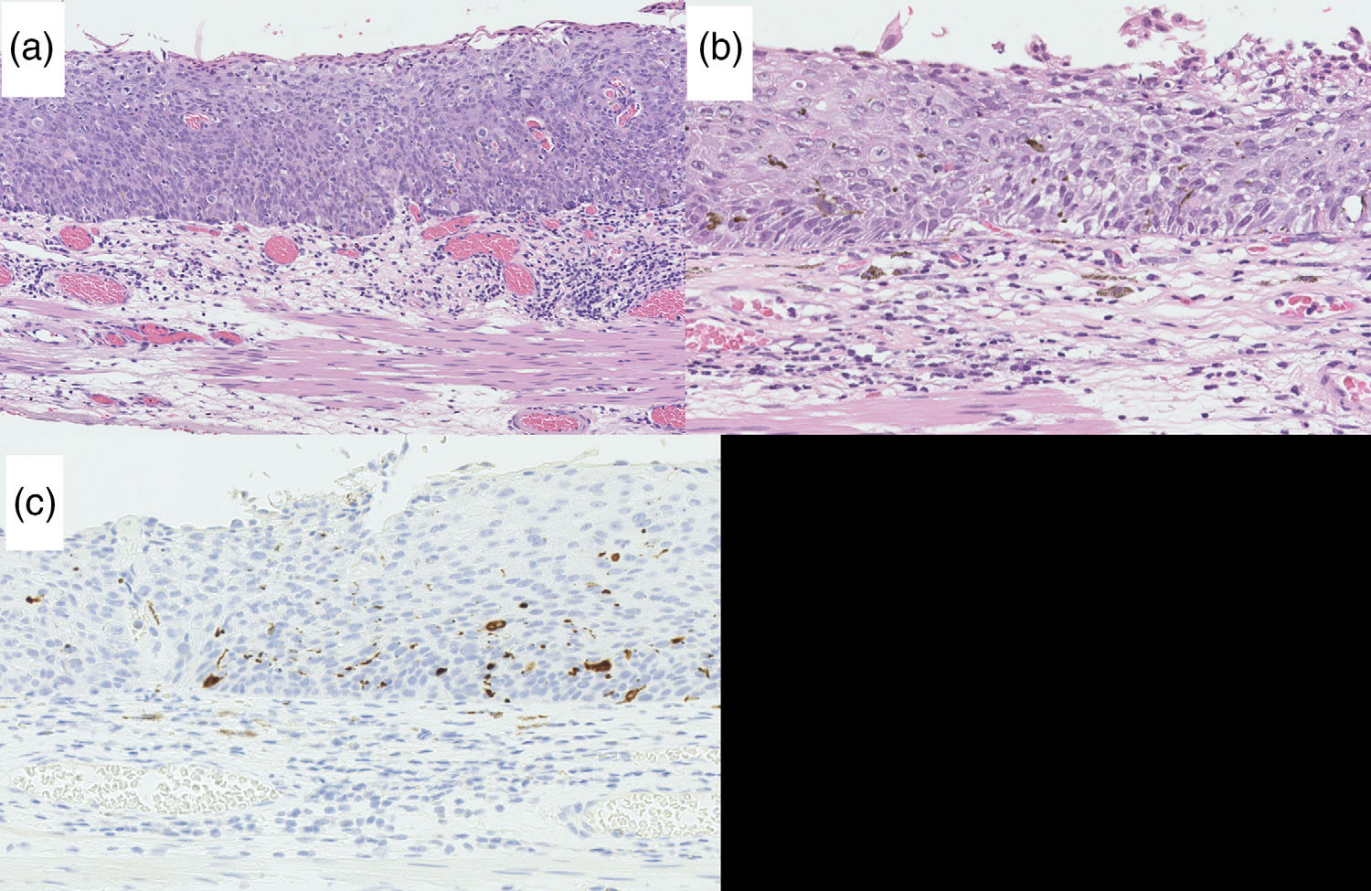
Histological and immunohistochemical findings of the resected specimen. (a) Hematoxylin–eosin staining of the iodine‐unstained region reveals squamous cell carcinoma with cellular atypia and irregular cellular arrangement, with slight invasion of the lamina propria (×200). (b) In the dark brown to black area, melanocytes were scattered within the epithelium, and macrophages phagocytosing melanin granules were also seen in the lamina propria (×400 HE). (c) The melanocytes in the epithelium were immunohistochemically Melan A positive (×400 Melan A).

An esophagogastroduodenoscopy performed at this hospital indicated a flat lesion (0–IIb) presenting with redness over two‐thirds of its circumference, which was located in the mid‐thoracic esophagus. We found three dark brown to black spots within the lesion (from the center toward the proximal side) with irregular and well‐defined borders (Figure 1a). The black spot shows slight thickening (Figure 1b). Narrow‐band imaging magnifying endoscopy indicated that the reddish lesion had type B1 vessels according to the Japanese Esophageal Society Magnifying Endoscopy Classification^2^ (Figure 1c). However, the vessels are not visible as dark brown or black spots. Lugol chromoendoscopy revealed that the entire lesion was 4 cm long and occupied two‐thirds of its circumference (Figure 1d). Based on these computed tomography and endoscopic findings, we suspected that the SCC lesion was confined to the lamina propria mucosa (Mt, 0–IIb, cT1aN0M0, and cStage 0). The lesion was accompanied by black spots with well‐defined borders that showed slight thickening. We suspected SCC with melanocytosis or malignant melanoma. We performed a complete resection via endoscopic submucosal dissection (ESD). We used a therapeutic endoscope of a GIF‐H290T (Olympus Medical Systems) with a short small‐caliber tip hood (DH28‐GR; FUJIFILM Medical Solution) attached to the ESD procedure. ESD resulted in a mucosal defect in four‐fifths of the esophageal circumference. A steroid solution containing 100 mg of triamcinolone acetonide was injected into the remaining submucosa in the area of the mucosal defect to prevent stenosis. There were no postoperative complications, such as bleeding or stenosis, and the postoperative course was excellent. The ESD specimen measured 42 × 30 mm^2^, with a flat lugol‐voiding lesion measuring 32 × 30 mm^2^ (Figure 2). Histologically, SCC with cellular atypia and irregular cellular arrangement was observed, with slight invasion of the lamina propria (Figure 3a). The extent of the SCC was consistent with that of a gross lugol‐voiding lesion. In the dark brown to black areas, melanocytes were scattered within the epithelium, and macrophages phagocytosing melanin granules were observed in the lamina propria (Figure 3b). Melanocytes in the epithelium were immunohistochemically positive for Melan A (Figure 3c), and no cellular atypia suggestive of malignant melanoma was observed. The final pathological diagnosis was SCC accompanied by melanocytosis. No vascular invasion was observed, and the resection margins were negative (32 × 30 mm^2^, 0–IIb, pT1a‐lamina propria mucosae, INFa, ly0, v0, pHM0, pVM0, and Stage 0).

## DISCUSSION

We encountered a rare case of superficial esophageal SCC with dark brown‐to‐black spots of melanocytosis that were difficult to differentiate from malignant melanoma. Excessive smoking and drinking are considered risk factors for esophageal melanocytosis and SCC.^3^

The endoscopic features of melanocytosis are flat dark brown lesions 3–5 mm in size with unclear borders.^4^, ^5^ In contrast, early‐stage esophageal malignant melanoma endoscopically shows a black spot with clear borders and a smooth surface.^4^, ^5^, ^6^, ^7^ Typical endoscopic findings of superficial esophageal SCC are reddish on white light imaging and brownish areas with dot‐like dilated vessels on narrow‐band imaging endoscopy. Thus, endoscopic features differ significantly between malignant melanoma and superficial SCC.^8^

In the present case, dark brown to black spots were observed in three parts of the superficial esophageal SCC lesion. One of three presented with a black spot with clear borders and slight thickening, consistent with early‐stage malignant melanoma.^4^, ^5^, ^6^, ^7^ The remaining two dark brown spots indicated melanocytosis. Esophageal melanocytosis is found in 25%–30% of malignant melanoma cases.^9^ In this case, we considered the possibility that the melanocytic lesions were concurrent malignant melanomas.

Although a previous study showed that histopathological diagnosis via biopsy is useful in differentiating malignant melanoma from melanocytosis in the esophagus, almost 20% of cases were misdiagnosed as poorly differentiated carcinoma.^10^ If only a small sample is obtained via biopsy or only surface tissue is obtained, it is highly likely that a definitive histological diagnosis cannot be made using the biopsy tissue alone. A previous study showed that endoscopic removal of small lesions was useful in the definitive diagnosis of malignant melanoma.^5^ It suggested that endoscopic removal could overcome the shortcomings of biopsy‐based diagnosis. Therefore, we performed ESD and achieved a definitive diagnosis of malignant melanoma.

Preoperative endoscopy revealed black spots with slight thickening, which were endoscopic findings similar to those of malignant melanoma. However, the final histopathological diagnosis based on ESD specimens was melanocytosis. Despite the well‐demarcated black spots with slight endoscopic thickening, a small number of melanocytes were histologically scattered in the basal layer. The reason for this was unknown. We hypothesized that SCC displaced all layers of the epithelium and infiltrated the lamina propria mucosae at the black spot, resulting in a slight thickening of the black spot area.

Here, we report a case of superficial SCC with melanocytosis that was endoscopically difficult to differentiate from malignant melanoma. When melanocytosis coexists with SCC infiltrating the lamina propria mucosae, melanocytosis may obscure the vessels, and SCC can cause a slight thickening of the mucosa layer, resulting in the endoscopic findings of malignant melanoma that showed black spots with slight thickening. In such cases, diagnostic treatment may be advisable because melanocytosis may misdiagnose the demarcation line of SCC and because it is difficult to distinguish it from malignant melanoma.

## CONFLICT OF INTEREST STATEMENT

There are no conflicts of interest to declare.
